# Effects of Organochlorine Pesticide Residues in Maternal Body on Infants

**DOI:** 10.3389/fendo.2022.890307

**Published:** 2022-06-09

**Authors:** Shi-Yu Qi, Xue-Ling Xu, Wen-Zhi Ma, Shou-Long Deng, Zheng-Xing Lian, Kun Yu

**Affiliations:** ^1^ College of Animal Science and Technology, China Agricultural University, Beijing, China; ^2^ Key Laboratory of Fertility Preservation and Maintenance of Ministry of Education, and Key Laboratory of Reproduction and Genetics of Ningxia Hui Autonomous Region, School of Basic Medical Science, Ningxia Medical University, Yinchuan, China; ^3^ National Health Commission of China (NHC) Key Laboratory of Human Disease Comparative Medicine, Institute of Laboratory Animal Sciences, Chinese Academy of Medical Sciences and Comparative Medicine Center, Peking Union Medical College, Beijing, China

**Keywords:** organochlorine pesticides, breast milk, lactation, infant, estrogen endocrine disruptors

## Abstract

There are many organochlorine pollutants in the environment, which can be directly or indirectly exposed to by mothers, and as estrogen endocrine disruptors can cause damage to the lactation capacity of the mammary gland. In addition, because breast milk contains a lot of nutrients, it is the most important food source for new-born babies. If mothers are exposed to organochlorine pesticides (OCPs), the lipophilic organochlorine contaminants can accumulate in breast milk fat and be passed to the infant through breast milk. Therefore, it is necessary to investigate organochlorine contaminants in human milk to estimate the health risks of these contaminants to breastfed infants. In addition, toxic substances in the mother can also be passed to the fetus through the placenta, which is also something we need to pay attention to. This article introduces several types of OCPs, such as dichlorodiphenyltrichloroethane (DDT), methoxychlor (MXC), hexachlorocyclohexane (HCH), endosulfan, chlordane, heptachlorand and hexachlorobenzene (HCB), mainly expounds their effects on women’s lactation ability and infant health, and provides reference for maternal and infant health. In addition, some measures and methods for the control of organochlorine pollutants are also described here.

## Introduction

Pesticides are one of the most widely used chemicals in the world and are divided into four chemical groups: organochlorine, organophosphate, carbamate and pyrethroid (1, 2). However, approximately 95% of pesticides may adversely affect non-target organisms during the application process (3). Organochlorine pesticides (OCPs) and their me7olites have toxic effects on higher organisms due to the presence of chlorine atoms in the compounds, as well as their low solubility and a tendency to preferentially partition into the lipophilic phase (4). Although some persistent organic pollutants (POPs) were banned or restricted decades ago (5), they are still often found in humans. They are resistant to microbial degradation, and their half-life ranges from several months to several years. They can be transported over long distances, and their presence can even be detected in the Arctic (6). They can biomagnify throughout the food chain and cause high concentrations in top predators including humans (7). OCPs are considered to be endocrine disrupting chemicals (EDCs) that can work at very low doses and are compounds that change hormones and homeostasis systems (8). They interfere with important life processes, such as sexual development, growth and reproduction, and the development of live fetuses (9).

OCPs can enter the human body through the absorption of the respiratory system, digestive system, skin and eyes (10). They are distributed throughout the body through metabolism, excretion and storage processes (11). Human exposure to OCPs, even at low exposure levels, can cause a variety of diseases. In addition to carcinogenic risks, neurotoxicity and genotoxicity, it can also have destructive effects on the endocrine, reproductive and immune systems (3, 12). Due to its lipophilicity and high persistence, OCPs accumulate in lipophilic human body parts, especially in fatty tissues and other lipid-rich tissues (13).

The breast is a hormone-dependent tissue. Its growth and differentiation require many hormones including estradiol, progesterone, and prolactin to coordinate (14–16). During pregnancy and lactation, breasts mainly secrete and store breast milk in response to the complex effects of hormones such as estrogen, progesterone and prolactin. OCPs can cause damage to the mammary glands and also affect the lactation ability of lactating women.

Breast milk provides almost all the necessary nutrients for babies under 6 months of age. It is a complete food that can improve and promote the growth and development of babies (17). About 60% of the lipids in breast milk come from the mother’s adipose tissue. Some OCPs stored in the mother’s adipose tissue will accumulate in breast milk and transfected to the baby through breastfeeding, which brings hidden dangers to the health of the baby (18). In addition, the developing fetus is at risk from OCPs *via* placental transfer (19). Therefore, breast milk is best used to represent the baby’s exposure to OCPs after birth. OCPs may affect infant anthropometric development (20), gut microbial function (21), and early childhood behaviour (22). OCPs exposure has a negative impact on infants’ neurological function (23). In addition, it has also been found that the OCPs in breast milk are closely related to cryptorchidism in male children (23). Compared with the after milk, the foremilk has a lower fat content. Compared with maternal serum, the content of DDT and HCH in post-milk is 80% higher (23). The lipid-adjusted concentration of OCPs will not continue to decrease during lactation, and there is no significant difference between the organochlorine content in colostrum and the organochlorine content in mature milk samples (24, 25). The residual level of persistent organic pollutants in breast milk may reflect the mother’s physical burden (26–28) and can be used to further examine the relationship between OCPs and maternal health and the possible health risks to breastfeeding offspring (29). Thus, the residue levels of POPs in breast milk and their related health consequences on infants are of high concern (30, 31).

This article reviews several main OCPs in the body fluids of pregnant women, such as dichlorodiphenyltrichloroethane (DDT), methoxychlor (MXC), hexachlorocyclohexane (HCH), endosulfan, chlordane, heptachlorand and hexachlorobenzene (HCB). On the one hand, it starts with the effect of estrogen endocrine disruptors on the mammary glands. On the other hand, it will provide some references for preventing maternal and infant diseases caused by OCPs.

## Main types of OCPs

Here we divided OCPs into 4 main sub-groups of OCPs viz: DDT and its analogues, HCH and its isomers, cyclodienes and HCB. Most OCPs can accumulate in breast milk. DDT, HCH and HCB are the main OCPs found in breast milk (32). The following is an introduction to several major OCPs (**Table 1**).

**Table 1:** Structure, physicochemical properties and biological effects of several organochlorine pesticides.

Name	structure	Mol. Wt	Water solubility (mg/L)	LogKow	Half-life (days)	Detection rate in breast milk	Role as xenoestrogens	Effects on Infant Health	References
Dichlorodiphenyltrichloroethane (DDT)	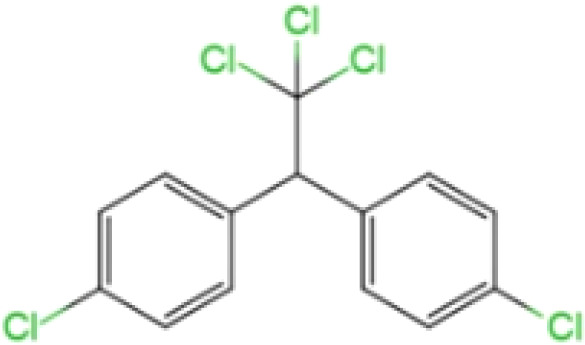	354.5	0.025	6.91	1460-3650	100% (Taiwan, China)	high binding affinity to ERα, ERβ and GPER	cause weight loss; affect neurodevelopment	(23, 33–38)
Dichlorodiphenyldichloroethylene (DDE)	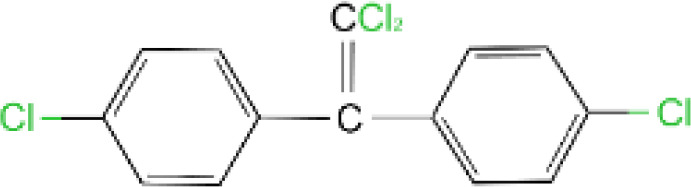	318.0	0.12	6.51	2064.8	nd	has weak estrogenic activity	cause weight loss; affect neurodevelopment	(34, 36–38)
Dichlorodiphenyldichloroethane (DDD)	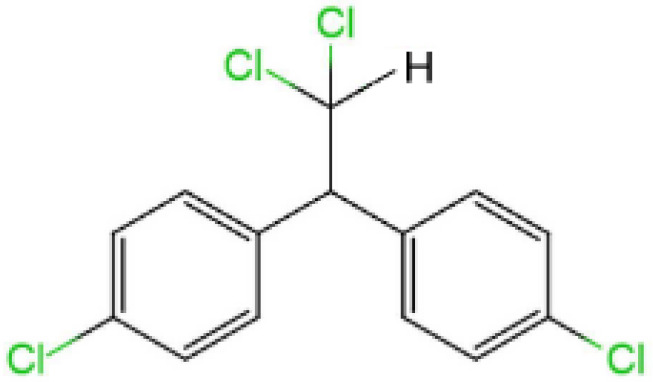	320.1	0.09	6.02	3800	nd	nd	affect neurodevelopment	(37, 38)
Methoxychlor (MXC)	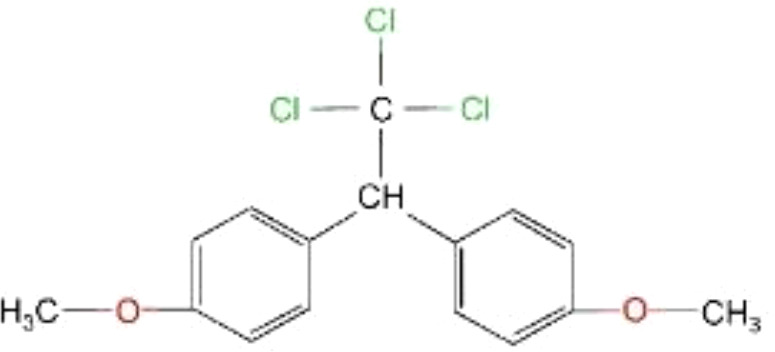	345.6	0.04	5.08	180	<50% (Taiwan, China)	bind to ESR1 and ERβ;act as an antagonist of AR	cause abnormal reproductive development	(23, 33, 38–43)
Hexachlorocyclohexane (HCH)	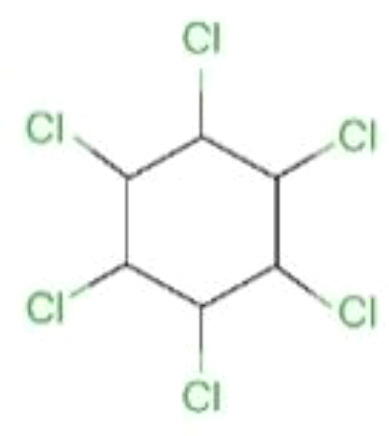	290.8	100	3.8	9490	36.4% (Jinhua, China)	has weak estrogenic activity	cause slow growth; affects the gut microbiome	(38, 44–47)
Endosulfan	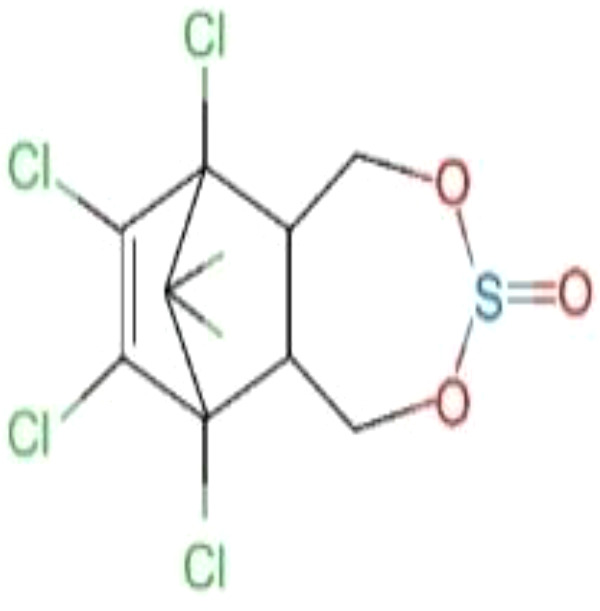	406.9	0.32	4.7	50-1095	<50% (Taiwan, China)	higher affinity with ERα	cryptorchidism in male infants	(23, 38, 48–50)
Chlordane	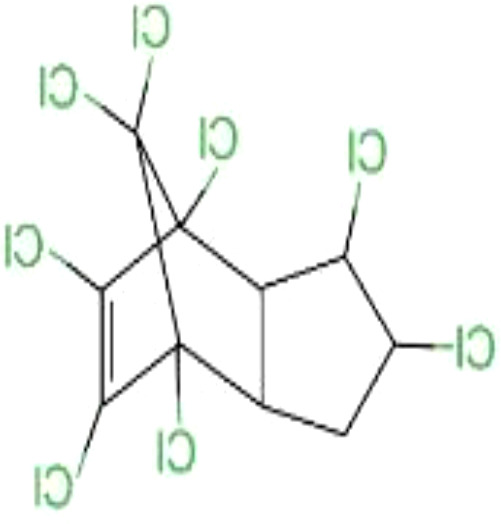	409.8	1	6.16	37-7300	nd	has weak estrogenic activity	affect neurodevelopment	(23, 38, 51)
Heptachlor	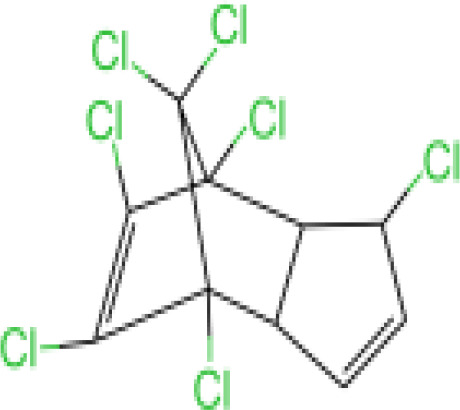	373.32	0.18	5.44	250	100% (Tiawan, China)	has weak estrogenic activity	decreased testosterone levels	(23, 38, 52)
Hexachlorobenzene (HCB)	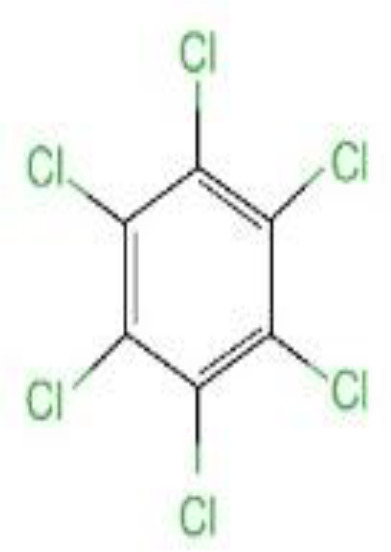	284.8	0.005	6.41	156-1563	83.6% (Jinhua, China)	activates AhR	lead to overweight; cryptorchidism in male infants	(42, 47, 50, 53–55)

“nd” means “not determined; ERα, estrogen receptor α;ERβ, estrogen receptor β; GPER, G-protein coupled estrogen receptor; AhR, aryl hydrocarbon receptor; AR, androgen receptor.

### Dichlorodiphenyltrichloroethane and Its Analogues

#### DDT and Its Isomers

Dichlorodiphenyltrichloroethane (DDT, 1,1,1-trichloro-2,2-bis(ρ-chlorophenyl) ethane), is a synthetic pesticide widely used for disease-vector control and agriculture. This compound can stimulate the release of gonadotropin-releasing hormones in a manner similar to that induced by estrogen interaction with estrogen receptors (ERs) (56, 57). DDT products usually consist of a mixture with 77% *p, p’*-isomers, 15% o, p’-isomers and traces of *o,o′*-DDT. The *o, p’*-DDT isomer is deemed to be the more estrogenic isomer and has a 100 times higher binding affinity for ERα than *p, p’* -DDT (33). Dichlorodiphenyldichloroethylene (DDE) is the main metabolite of DDT and can be considered as an environmental contaminant (58). The half-life of DDT and its main metabolite (DDE) in humans is at least 5 years, the longest can even reach 10 years (34, 35). Humans are primarily exposed to *p,p’*-DDT and *o,p’*-DDT through direct exposure to pesticide application and dietary exposure, whereas the source of *p,p’*-DDE exposure depends on whether *p,p’*-DDT exposure is effective. In addition, *p,p’*-DDE can also be directly exposed through diet and other aspects. The higher detectability and levels of *p,p’*-DDE in humans may be due to the longer half-life of this compound in the environment (36). Dichlorodiphenyldichloroethane (DDD) is also an important metabolite, and DDT can also be reductively dechlorinated to DDD under reducing conditions (59). Reductive dechlorination (RD) is also the main mechanism by which microorganisms convert *o,p’*-DDT and *p,p’*-DDT isomers to DDD (4). Effects of DDT include liver and central nervous system toxicity, estrogenic and antiandrogenic effects, and possible carcinogenicity (60). Some epidemiological evidence suggests that DDT and its metabolites exposure increases preterm and small-for-gestational-age births and shortens lactation (61, 62).

#### (DDT, 1,1,1-trichloro-2,2-bis(ρ‑chlorophenyl) ethane)

Because DDT was banned long ago, MXC emerged as an alternative to DDT (63). MXC, a structural analogy of DDT, has been found in human tissue samples and breast milk (23). Its half-life is shorter than that of DDT, about half a year (38). MXC has a chlorinated double ring structure. It can bind to ER. The purity of MXC has an effect on its binding ability on ER, and there are obvious differences in the binding of MXC with different purities on ER. 95% MXC has a weak affinity for ER, while 99% MXC has no competitive effect on ER (39). Its metabolite (2,2-bis-(p-hydroxyphenyl)-1,1,1-trichloroethane) (HPTE) can bind to ESR1 (Estrogen Receptor 1) and ERβ and has antiestrogenic activity. Also acts as an antagonist of androgen receptor (AR) (40, 41). It adversely affects female fertility, early pregnancy and uterine development (63).

### Hexachlorocyclohexane and Its Isomers

Industrial grade HCH is highly stable and resistant to degradation, and has been used to kill parasites such as mosquitoes for malaria or typhoid control for a long time (64, 65). Although these compounds were banned in the 1990s, they are still used in some developing countries. The production of such saturated cyclic compounds leads to the production of several isomers with different spatial arrangements of chlorine atoms around the cyclohexane ring. There are five stable isomers, including α: 60%-70%, β: 5%-12%, γ: 10%-12%, δ: 6%-10% and ϵ: 3%-4% (66). HCH can persist in the environment for a long time, for example, the predicted half-life of γ-HCH in water is 191 days (44). The average half-life of γ- and α-HCH around the Great Lakes region is about 3 to 4 years (38). It was later found that only γ-HCH (also known as lindane) has insecticidal activity (67). Lindane production process is very inefficient, producing 1 ton of lindane produces about 8-12 tons of HCH waste consisting of α-, β-, δ- and ϵ-isomers (68). Although only the γ-HCH is insecticidal, industrial grade HCH is widely used as an inexpensive and effective insecticide in developing countries (67).

In the environment, some bacteria can slowly isomerize lindane into α-, β- and δ-HCH, mainly under anaerobic conditions (69). The α-, β- and δ- isomers are in many ways more problematic than lindane itself. Of all isomers, β-HCH was noted most commonly, which is a result of its high stability and persistence. The β-isomer is usually present in higher concentrations than those of α- and γ-HCH, which metabolize into β-HCH in the human body (66). Among the isomeric forms of HCH, β-HCH has a more stubborn chemical structure, which makes it more resistant to biodegradation. Once ingested or inhaled, it leaves the body very slowly due to its higher lipophilicity. Therefore, in the case of long-term exposure, β-HCH is more toxic than the other isomers and has a significant adverse effect on fetal growth (70).

### Cyclodienes

#### Endosulfan

Endosulfan (6,7,8,9,10,10-hexachloro-1,5,5a,6,9,9ahexahydro-6,9-methano-2,4,3-benzodioxathiepin-3-oxide) consists of α- and β-isomers in a ratio of approximately 7:3 (42, 71). This insecticide is used to control pests on fruits, vegetables and tea, as well as non-food crops such as tobacco and cotton. It is also used as a wood preservative. Because of its high efficiency, low cost and environmental stability, it is widely used mainly in developing countries (71). Endosulfan is semi-volatile and relatively persistent. It has a half-life of 6 years. Once endosulfan is released into the environment, it undergoes a transformation process that regulates its presence in soil, sediment, water, and biota (48). In addition, he also has a higher affinity with ERα (49), long-term effects on female fertility and differentiation of uterine function in early pregnancy (71).

#### Chlordane

Chlordane is a cyclopentadiene-derived insecticide that was once used to control termites and borers. Industrial chlordane is a complex mixture of structurally related chemicals, including cis-chlordane, trans-chlordane, and heptachlor (72). Due to its toxicity and persistence, it can remain in the soil for up to 20 years, so chlordane and its related compounds can still be detected in the environment (51). Chlordane initially accumulates in the liver and kidneys and then redistributes to adipose tissue (73). Chlordane has toxic effects on the nervous system, causing headaches, confusion, convulsions, and even fatal symptoms (74).

Many cyclopentadiene pesticides are chiral and exist as non-superimposable mirror-image pairs of enantiomers (72, 75). Chlordane mixtures are racemic (they contain two pairs of each chiral component 1:1 mixture of enantiomers). Chiral organochlorine compounds can cause enantioselective and even enantiospecific biological effects (76, 77).

#### Heptachlor

Chlorinated cyclodiene heptachlor was registered in 1952 as an agricultural and household insecticide, mainly for the control of termites and fire ants (78). *In vivo* studies have shown that heptachloro epoxide is the major metabolite. In just a few hours, about 20% of the heptachlor in the body is degraded to epoxy heptachlor. Formed as a product of a mixed-function oxidase system, 1-hydroxychloride is the major soil metabolite (78). In breast milk, higher levels of heptachlor residues are present. Heptachlor was detected in 100% of 55 breast milk samples in a study in southern Taiwan (23). Heptachlor exposure has been found to be associated with neurodegenerative and neurobehavioral disorders such as Parkinson’s disease (79) and depression (80).

### Hexachlorobenzene

HCBs are widely present in the environment around us, mainly used as a fungicide. At present, HCB is no longer used as a fungicide, but it is a by-product produced in the commercial chlorination process (81). It adheres strongly to soil and degrades very slowly (53). It is poorly soluble in water, and once there, it accumulates in sediments (54). Its half-life is about 2 years in air and 6 years in water and sediments (82). The main route of exposure of the general population to HCB is through foods such as fatty fish. HCB is a hormone disruptor as a weak ligand for dioxin-like compounds and the aryl hydrocarbon receptor (AhR) (83). At the molecular level, HCB first activates the AhR. Subsequently, AhR will form heterodimerization with AhR nuclear transporter (ARNt) and bind to specific gene regulatory sequences of xenobiotic response element (XRE) (42, 84). There are many adverse effects of long-term exposure to HCB, including neurological symptoms, immune disorders and thyroid dysfunction (82).

## Factors Influencing the Level of Organochlorine Pesticides in Breast Milk

**Figure 1:**
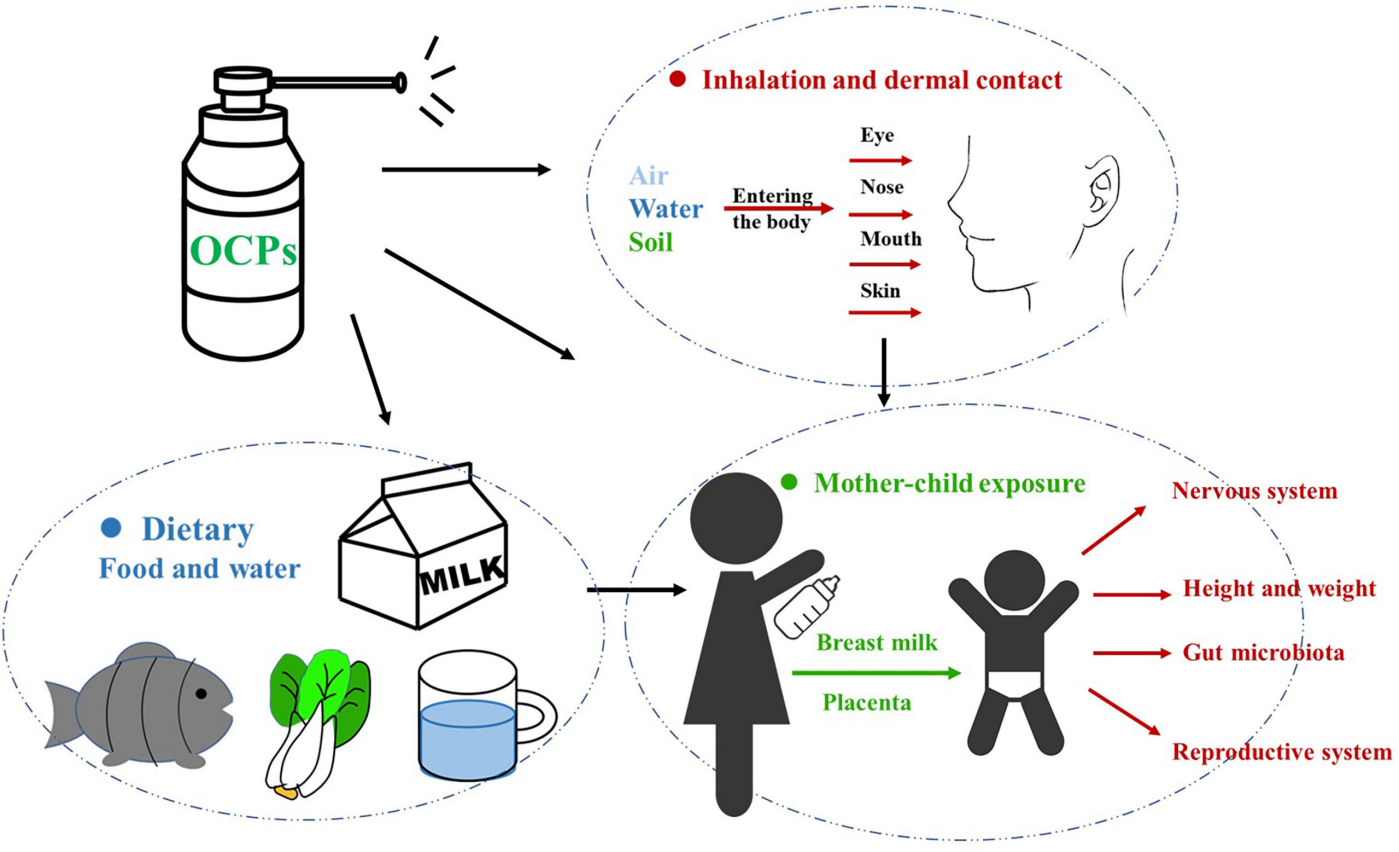
Mothers before and after pregnancy can be directly or indirectly exposed to OCPs, which are passed to the fetus/infants through the placenta/breast milk, causing damage to the growth and development of the babies. OCPs, organochlorine pesticides.

The pollution rate of humans mainly depends on individual exposure and accumulation, and is affected by local soil and air pollution, diet, exposure duration, age, metabolic elimination ability, and breast milk production (13, 85) (**Figure 1**).

### Food

Eating habit is an important factor that affects the level of OCPs in breast milk and women’s related risks. Dietary exposure accounts for more than 90% of the total body burden of organochlorines (32). OCPs tend to slowly bioaccumulate in the food chain, such that they are eventually ingested by women and enriched in adipose tissues; in these tissues, OCPs can persist for a long period of time (86). The highest residues of these compounds are found in fish, meat, poultry, eggs, milk and dairy products, as well as vegetable oils, nuts, avocados, sesame or olives (87–89). For example, fruits and grains are the main sources of DDE (90). The level of DDT in breast milk is closely related to the consumption of animal-derived food and aquatic food (91). Of these, bioaccumulation of organic compounds in fish and other animals and their products (meat and dairy) contributes to substantial exposure to OCPs in humans through ingestion due to their high fat solubility (92). It has been observed that vegetarians (i.e. those who consume vegetables, fruits and grains without animal products) have significantly lower levels of oral contraceptives compared to those who consume animal products (93). However, it is not to say that vegetables do not cause the accumulation of OCPs. In one study, food preferences in vegetables were found to be correlated with HCB, HCHs (ΣHCHs, β-HCH, γ-HCH), *p,p’-*DDE and heptachlor (Σ heptachlor and trans-heptachlor epoxides) in breast milk concentrations were significantly positively correlated (94). In addition to contamination of dairy products through the animals themselves, a major source of dairy contamination may be the presence of OCPs such as HCH and DDT in dry and green feeds (19).Untreated agricultural wastewater (including pesticides, etc.) released into the water may cause the accumulation of organochlorines, leading to pollution of the aquatic ecosystem (95). At the same time, it also led to the pollution of aquatic organisms such as fish and shrimp. Humans feed on these, and as the food chain accumulates, they are ultimately the most harmed by OCPs. Fish consumption has been found to be an important factor leading to elevated OCP levels in human breast milk. β-HCH, δ-HCH, *p’-*DDT, *p’-*endosulfan, HCB, *p,p’-*DDT, cis-chlordane and methoxychlor in breast milk of fish-eating mothers are generally higher than that of mothers who do not eat fish (32). Studies have also found that heat treatment of fish, especially grilling, can reduce the level of OCPs residues in fish (95). The reason for this may be that the heat treatment causes changes in the lipid content of fish meat, which eventually leads to a decrease in OCP levels.

### Region

Residents of developed countries (North America and Western Europe) have lower levels of OCPs in breast milk than those of developing countries, which may reflect differences in exposure (96). The reason for this may be that OCPs have long been banned in most developed countries, but they are still widely used in many third world countries because they are cheap and readily available. Or the time of ban in developing countries is later than that in developed countries. It was found heptachlor-epoxide isomer B in breast milk collected between the second and eighth weeks of lactation in samples from rural and urban Australia. The average lipid of samples from rural areas was 16.7 ng/g lipids, while the average in samples from urban areas was 2.21 ng/g lipids (69, 97). This may be related to the use of pesticides in rural areas. In two surveys of the levels of persistent OCPs in breast milk of Chinese mothers, significant differences were found between rural and urban areas in 1998, while the results in 2011 showed little difference. Presumably due to the rapid urbanization and industrialization of rural areas, this difference has been reduced (98, 99). However, some studies have found that the place of residence has nothing to do with the concentration of DDT or HCH in breast milk (69). Studies have also found that mothers who work or live near industrial plants or where factories used to be more susceptible to contamination (100).

### Physiological Factors

Some studies have found that the concentration of OCPs (DDT, diphenylether, HCB, βHCH, dieldrin and MXC, etc) in breast milk increases with the mother’s age (30, 101), but some studies have not found this situation (69). Older mothers, who have longer lifetime exposures, may transfer more OCPs to their first infants through breastfeeding than mothers of younger maternal age. Therefore, first births from older mothers may be at higher risk for OCPs (102). The increase in the concentration of OCPs with age may be caused by eating habits. On the other hand, pesticides are resistant to metabolic processes in the body, and they will bioaccumulate with age (103, 104). And this lack of correlation may be due to the short exposure period or the disappearance of these organics in the environment (69). Because the breast milk of prenatally exposed mothers may have higher levels of organochlorine, prenatal exposure is probably the most critical window of exposure, more critical than any other period of postnatal life (105). Studies have found that women’s weight before and after pregnancy affects changes in the concentration of organochlorines in breast milk (101, 106), residues in breast milk are due to accumulation of fat. A study found that women with low gestational weight gain retained higher levels of contaminants in their colostrum (107). This is because increases in body weight and fat have the effect of diluting OCPs concentrations (107). In addition, insufficient levels of maternal fat may lead to higher rates of mobilization of maternal fat stores in the last trimester of pregnancy (107), which may trigger the release of oral contraceptives into the bloodstream and ultimately into colostrum and breast milk. The colostrum of low gestational weight mothers transmits more contaminants to the baby (108). Some OCPs, such as cis- Chlordane and γ-HCH, are also related to menstrual characteristics, hormone intake, and treatment of infertility (109). Women with irregular menstrual cycles have observed higher levels of dioxins and polybrominated diphenyl ethers (110, 111). This also shows that OCPs may interfere with hormonal balance, thereby destroying menstrual characteristics. Due to differences in metabolism between individuals, large inter-individual differences in pesticide concentrations in breast milk were also found (50).

## The Health Effect of OCPs on Mother and Baby

### The Effect of OCPs on Lactation

OCPs, including DDE, interfere with the mother’s ability to lactate, possibly because of its estrogenic properties (112). In women, the endocrine system is responsible for hormonal balance and reproductive potential; chemicals that share similar structures with estrogen, act as agonists/antagonists of ERs, or induce ER-mediated signalling can be considered estrogen endocrine disruptors (EEDs, also called xenoestrogens). The expression of ERα and ERβ was found in both mammary epithelial cells and mesenchymal cells. ERα is essential for mammary gland development and lactation; ERβ is more involved in lobular acinar development (113). Because of its aromatic A ring and C3-hydroxyl that provide properties of both hydrogen-bond donor and acceptor, 17β-estradiol (E2) can interact with Arg394 and Glu353 in ERα or with Arg346 and Glu305 in ERβ (114, 115). Moreover, unlike testosterone, E2 lacks a C19 methyl group, which ensures that the interface between ring A and B is flat, resulting in closer contact with ERs. The C17-hydroxyl on the D ring is of equal importance during binding of E2 to ERs; it exhibits stable contact with His524 in ERα and His475 in Erβ (114, 116, 117). Presumably, chemicals containing two rings properly spaced (i.e., in the manner present in E2) can bind to ERs and disrupt normal estrogenic physiology. But in most cases the relative binding affinity (RBA) was at least 1000-fold lower than that of E2 (118). While most OCPs are generally classified as “weak” estrogens, they act “additively” to endogenous estrogens, and when ingested in sufficient quantities, they can affect the endocrine system (119, 120). OCPs can act as estrogen ligands and cause damage to female reproductive physiology, especially lactation. During early pregnancy, the ductal system dilates into adipose tissue in response to an increase in estrogen. Estrogen also stimulates the pituitary gland, leading to increased prolactin levels (121).. Later in pregnancy, the mammary glands are fully developed to produce milk due to prolactin stimulation. High levels of circulating prolactin and estrogen can increase the surface area of the glands and ducts in the breast, impairing milk synthesis. Only at term, and when estrogen begins to decline, does prolactin begin to boost milk synthesis. Weak but persistent estrogen analogs like OCPs such as DDE interfere with milk synthesis, causing early weaning (122). In addition, OCPs can compete for prolactin receptors (PRLR), thereby inhibiting the lactation effect of PRL.

Prolactin (PRL) is generally regarded as a pituitary hormone that stimulates and maintains milk secretion (123). Specifically, PRL induces cell proliferation by activating the cyclin D1 promoter through the JAK2/STAT pathway, promoting cell growth and differentiation of alveolar cells in the mammary gland (124). The secretion of PRL is also related to cell proliferation, cellular immune response and hormonal regulation of parental behavior (125–127). Excessive stimulation or inappropriate stimulation at the developmental stage or reproductive cycle stage may inhibit the secretion of PRL, thereby destroying normal reproductive function (128). The rapid response to EED involves the mobilization of various second messengers, such as cyclic adenosine monophosphate (cAMP) and calcium ions (Ca2+), and G-protein coupled estrogen receptor (GPER) can mediate these rapid effects to activate various signaling cascades (129). The response of Ca^2+^ to extracellular stimuli can lead to cell movement, intracellular and extracellular signal transduction processes, and rapid secretion of hormones through exocytosis, among which prolactin can be secreted in this way (130) Studies have found that several OCPs, including DDE and endosulfan, can quickly induce Ca^2+^ influx at very low concentrations (picomolar to nanomolar), leading to PRL secretion and undermining endocrine function (128). Studies have found that HCB treatment significantly reduces plasma prolactin concentrations, which may lead to reproductive changes. But it cannot be determined whether HCB directly affects the glands or reduces the release of prolactin from the pituitary gland by changing the concentration of dopamine (131). In addition, the level of oxytocin has a certain effect on new-born babies. Some studies have also found that new-borns with lower levels of prolactin in cord blood have an increased risk of respiratory distress syndrome than new-borns with higher levels of prolactin (132, 133).

Gonadotropin-releasing hormone (GnRh) is a neurohormone secreted by the hypothalamus that regulates reproduction in both sexes through the secretion of pituitary gonadotropins. Expression of GnRH and its receptors has also been found in various peripheral tissues (134). GnRH can promote the pituitary gland to secrete luteinizing hormone (LH), follicle-stimulating hormone (FSH) and prolactin (PRL). Prolactin acts on the mammary gland to promote mammary gland development and milk production. ANXA5 is a member of the annexin family of proteins, grouped by its structural similarity and affinity to calcium and phospholipids (135, 136). ANXA5 is thought to be involved in GnRH receptor signaling (136), and reduction of PRL increases GnRH in breast tissue, leading to ANXA5 expression, which enhances epithelial cell apoptosis and reduces milk production and weight (135).

**Figure 2:**
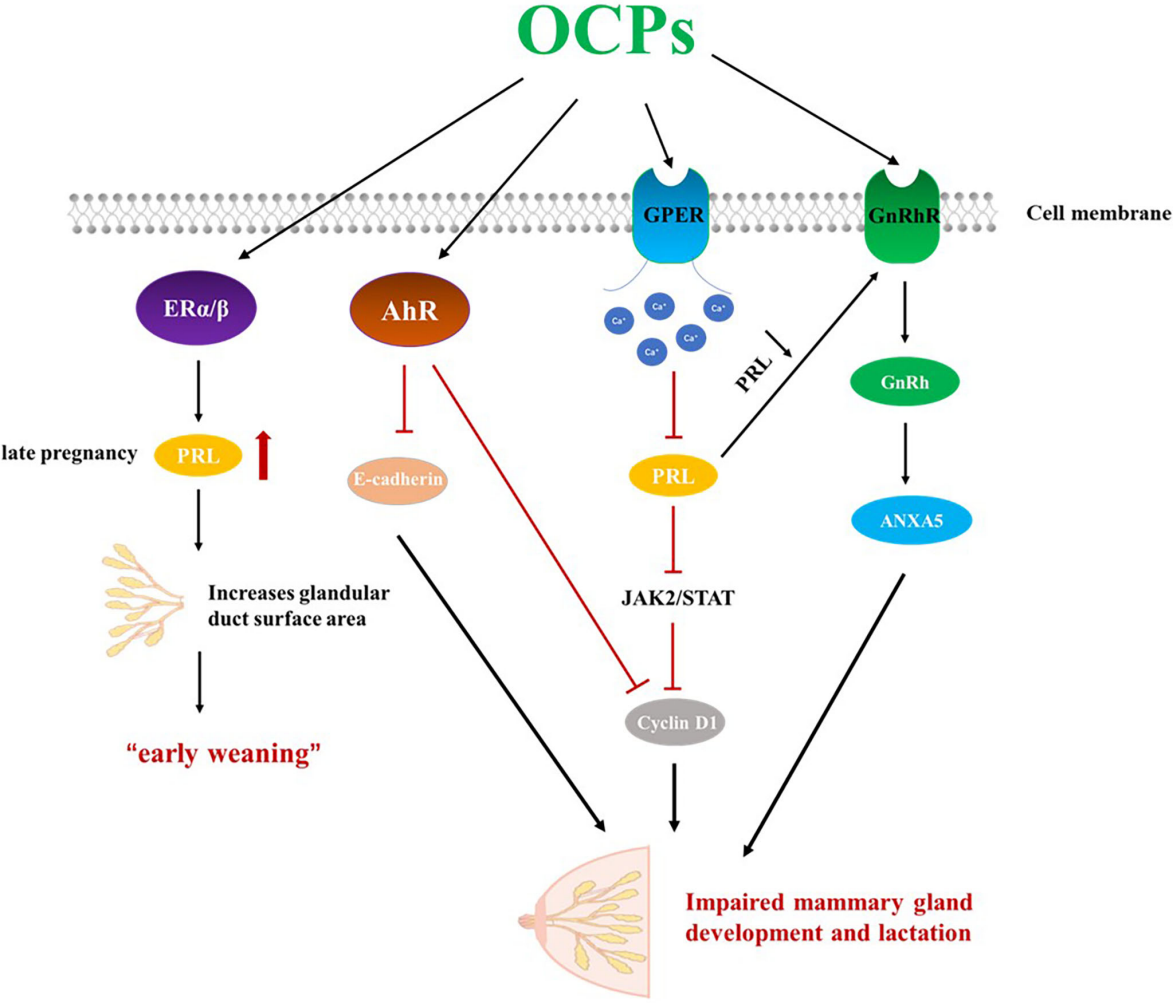
OCPs can act as estrogen endocrine disruptors and impair lactation. OCPs, organochlorine pesticides; ERα, estrogen receptors α; ERβ, estrogen receptors β; GPER, G-protein coupled estrogen receptor; AhR, aryl hydrocarbon receptor; GnRhR, Gonadotropin-releasing hormone receptor; GnRh, Gonadotropin-releasing hormone; PRL, Prolactin.

Another target pathway by which OCPs affect lactation is through activation of the AhR. Activation of AhR, an environment-aware transcription factor, leads to severely impaired mammary gland differentiation during pregnancy, including reduced ductal branching, poor alveolar structure formation, blocked milk protein expression, and impaired lactation capacity (137). E-cadherin is a cell adhesion molecule that is directly involved in the development and differentiation of epithelial cells in various tissues including the mammary gland (138). In late pregnancy, activation of AhR reduces the expression level of E-cadherin in mammary cells, thereby affecting mammary gland development. Defects in mammary gland differentiation and lactogenesis result from direct effects on mammary epithelial cells (MECs). AhR-mediated reduction of cyclin D1 provides a possible mechanism for reducing pregnancy-related MEC proliferation (137) (**Figure 2**).

### Effects of OCPs in Breast Milk on Infants

Some evidence suggests that fetuses and infants may be more sensitive to OCPs than adults (139). Because children’s metabolic pathways are immature, their detoxification is much poorer than in adults, especially during the fetal period and the first year of life. Additionally, developmental processes during these periods are also more susceptible to disruption due to their higher body surface area relative to their body size (140). In general, OCPs with molecular weight <800 D present in plasma enter breast tissue by passive transport (141). Early life exposure through breastfeeding significantly increases the physical burden of oral contraceptives in children and is thought to be a major determinant of blood levels in children before age 7 years (25, 142) (**Figure 1**).

#### Effects on Neonatal Growth and Development

Breastfeeding is very important for the health effects of postpartum exposure to infants. OCPs will affect the growth and development of babies. In Norway, there is an association between an increase in HCB in breast milk and a decrease in birth weight (BW), crown heel length (CHL) and head circumference (HC) (143). There is a correlation between higher β-HCH in female breast milk and lower bio-organic weight (144), Early exposure to β-HCH is also associated with slower growth (45). In addition, residual DDT in breast milk also has a significant impact on fetal growth (BW, CHL, HC and CC) (20). OCPs are associated with lipogenesis and weight gain, and they may increase the risk of childhood obesity (145). Prenatal exposure to HCB increases the risk of being overweight in children under the age of 6 (55). OCPs in breast milk were positively associated with rapid growth and obesity in the first 6 months of life (146). However, studies have also found that *o,p’*-DDT and *p,p’*-DDE are associated with decreased birth weight at a given *p,p’*-DDT exposure level (36).

#### Effects on the Development of the Nervous System

Exposure to OCPs in breast milk negatively affects infants’ neurological function. At present, the exact mechanism of the negative effects of EDCs such as OCPs on the neurodevelopment of infants and young children is unclear. However, several possible mechanisms can be proposed. First, the neurotransmitter γ-aminobutyric acid (GABA) is a key mediator of neural development. It acts on all neuron types through GABA receptors located on adjacent neurons to promote neurogenesis, neuronal migration, and synapse formation, among others (147). The primary goal of OCPs such as endosulfan is to inhibit GABA receptors, which can affect multiple aspects of neuronal circuits in the frontal cortex, resulting in impaired cognitive function and behavioral deficits (148). In addition, OCPs exposure disrupts thyroid hormones with adverse consequences on developing brain nerves (149). Finally, disruption of calcium signalling, activation of peroxisome proliferator-activated receptors (PPARs), and lipid metabolism can also be used to explain the adverse neurodevelopmental effects of some OCPs (150). DDT and its metabolites DDE and DDD will pass through the placenta and contaminate breast milk, causing toxicity related to infant neurodevelopment (37). Studies have found in samples of breast milk consumed by 18-month-old infants that high levels of chlordecone can cause neuronal damage, such as poor fine motor scores in boys (151). After adjusting for confounding factors such as maternal age, pre-pregnancy body mass index (BMI), and baby gender, Kao et al. (23) used a logistic regression model and found that the breast milk levels of 4,4′-DDT were associated with cognition and language, while trans-Chlordane were associated with socioemotional scores. Significantly negative correlation. In addition, the amount and duration of breastfeeding may be a covariate related to infant neurodevelopment when breastfed infants are exposed to OCPs postpartum (23). studies have shown that exposure to heptachlor is associated with disruption of the dopamine system (152). A study in Japan also found a significant negative association between low prenatal exposure to cis-heptachloroepoxide and Mental Development Index (MDI) at 18 months of age (153). In addition, heptachloroepoxide had the highest negative correlation with the adaptive behavior scale (23).

#### Effects on the Gut Microbiome

The gut microbiota (GM) is essential for the development and maturation of the gastrointestinal tract (154, 155). Early disruption of microbial communities may have lifelong health consequences. The developing infant’s gut microbiome is directly exposed to OCPs-contaminated breast milk. There are many studies on whether OCPs in breast milk affect the composition and function of the infant’s gut microbiome (21, 156). HCH alters the colonization of the infant gut by altering the microbial composition in human colostrum (46). In addition, studies investigating whether environmental pollutants (including organochlorines) in breast milk affect the homeostasis of the infant gut microbiota at one month revealed differences in the abundance of some Firmicutes strains (21).

#### Effects on Reproductive System Development

As an endocrine disruptor, OCPs can adversely affect the reproductive system of infants, which has been demonstrated in many studies (157, 158). Breast tissue is not fully developed at birth, so hormones are critical for its development at this stage (159). Furthermore, cells are in a state of rapid growth and differentiation during this period. Exposure to OCPs at this stage may increase breast cancer incidence (160). Early exposure to OCPs also affected normal prostate function and structure, with a notable combination being DDT and its metabolite DDE. Low amounts of DDT were able to inhibit PSA at the mRNA and protein levels (161). Congenital cryptorchidism is a genital malformation disorder that occurs in newborn boys and is associated with decreased semen quality and an increased risk of testicular cancer (162). There is an association between congenital cryptorchidism and some persistent OCPs(*p,p*′-DDT, *p,p*′-DDE, *p,p*′-DDD, *o,p*′-DDT, HCH (α, β, γ), HCB, α -endosulfan)present in the mother’s breast milk, which can adversely affect testicular descent in boys (50, 163).

### Effects of OCP on the Baby Through the Placenta

Fetal exposure to OCPs and other POPs is primarily through placental transfusion (99). The placenta is an important barrier for fetal protection during pregnancy. The placenta produces human chorionic gonadotropin (hCG), which is involved in the exchange of gases, nutrients, and waste between mother and fetus (164). Estimated levels of OCPs in breast milk, placenta, and fetal tissue were 16.7, 10.1, and 5.3 (ng/kg lipid) (66, 165). Despite the lower burden, transplacental exposure of the developing fetus remains important for the child’s physical development and cognitive functions, even more so than postpartum exposure through breast milk (166). Organic chloride pollutants have high affinity for hydrocarbon (Ah) receptors (167). A combination of strongly induced cytochrome P4501A1 (CYP1A1) genes encoding cytochrome P-450 1A1 enzymes involved in the metabolism of organochlorines (168). The metabolism of organochlorine in the body produces a large amount of reactive oxygen species, and the antioxidant system cannot eliminate these reactive oxygen species, thereby damaging the DNA chain and affecting mitochondrial function. CYP1A1 is involved in human placental metabolism; abnormal expression of this enzyme may disrupt placental detoxification mechanisms (164, 169). During pregnancy, OCPs can enter the maternal circulation and reach the placenta (170). These substances can interfere with the placenta, such as the production and release of hormones and enzymes, the transport of nutrients, and the production of waste, and then disrupt fetal development and the final stages of placental life (171). Higher concentrations of β-HCH in cord blood increase the risk of preterm birth. In addition, researchers noted that serum γ-HCH levels were positively associated with habitual miscarriage in women (172, 173). MXC is thought to be a testosterone trigger, and exposure to OCPs during pregnancy may disrupt maternal hormones that regulate offspring sex, resulting in higher rates of boy production (2, 174). Furthermore, MXC crosses the placenta and induces abnormal reproductive development (43). Prenatal exposure to alpha endosulfan and heptachlor increases estradiol and sex hormone binding globulin (SHBG), thereby reducing testosterone levels in infants at birth (52).

## The Method of Prevention

Overall, in the past 30 years, OCPs in breast milk have decreased in many countries. For example, in the case of DDT, since the 1990s, lipids have dropped from 2000-32,000 ng/g lipid to 0.1 ng/g lipid (69). However, the harm of OCPs to mothers and babies is still very serious, and it is very necessary to find suitable prevention methods.

At present, although most countries and regions have explicitly banned the use of OCPs, in some developing countries, such as African, Asian and Latin American countries, there is no or only partial control, and polychlorinated biphenyls and other chemicals can still be found. Concentrations are high (175). The use of pesticides can be dealt with from a socio-economic perspective, and an explicit prohibition can be made, if necessary, which is the most fundamental means to solve the problem of OCPs pollution. Since OCPs are difficult to degrade, they still circulate in water, sediment and soil, affecting the health and safety of humans, especially mothers and infants.

The governance of OCPs in environments such as water and soil is necessary. Numerous techniques are available to treat existing organochlorine compounds in the environment, involving physical, chemical, and biological methods such as adsorption, oxidation, catalytic degradation, membrane filtration, and biological treatment (38). Biological treatment is an ideal approach, but due to the stubborn structure of OCPs, few specific bacterial and fungal species can degrade them, and finding them is cumbersome (176, 177). Currently, many researchers focus on the development and integration of electrochemical oxidation techniques. The main advantages of this approach are that no chemicals or other products need to be added, and the process can be easily connected to renewable energy sources (178). However, when dealing with large volumes of wastewater, the size of equipment and devices increases with energy consumption and waste generation, in addition to unavoidable side reactions and mass transfer limitations (179, 180). The use of nanoparticles to remove this type of contaminant has also been well studied (38). Lindane was removed from aqueous solutions using FeS nanoparticles stabilized by biopolymers (181). In addition, most nanomaterials are semiconductive in nature and can easily photocatalyzed the degradation of OCPs. The conditions of the photodegradation process simulate real environmental conditions and can be applied to soils on a large scale.

Soybean isoflavones are a kind of plant estrogen, extracted in soybean, mainly including genistein and daidzein. There is an interaction effect between OCPs and isoflavones (182). Several studies suggest that flavonoids such as soy may be added to infant supplements as protective molecules to counteract the effects of OCPs in infants (161). However, studies have found that genistein mixed with common pollutants (DDT, DDE, endosulfan) can affect the mammary gland (183). Conversely, prepubertal exposure to genistein prevents the development of breast tumors (184). Melatonin (N-acetyl-5-methoxytryptamine), which protects against cellular damage caused by reactive oxygen species (185), has some preventive functions. Studies have found that melatonin reduces lesions in epithelial compartments, tubular atrophy and vascular congestion (186), and has a protective function in the prostate. Green tea is a non-oxidized and non-fermented form of tea that contains several polyphenols, including green tea catechins. In preclinical studies, green tea extract and polyphenols have been shown to reduce the toxicity of DDT (187).

In addition, several studies have shown that cooking food in different forms can effectively reduce the content of organochlorine. Research on animal meat samples such as cows, goats and chickens. Among the raw meats, beef samples were found to have the highest levels of contamination and chicken samples to have the lowest levels of contamination. Subsequent decontamination studies have shown that cooking is the best option for reducing pesticide levels in raw meat samples. Cooked chicken is the safest food to eat (188). Heat-treated fish can also effectively reduce the level of OCPs residues (95). Milk is an ideal solvent for fat-soluble OCPs. Comparing the levels of OCPs in fresh raw milk and pasteurized milk, the levels found in raw milk were higher than pasteurized milk, and the results verified the efficient effect of heat treatment on the degradation of OCPs (189).

Finally, Information and education can be provided to the public to minimize exposure to possible OCPs, educate and awareness among farmers about the harmful effects of OCPs and how to handle them properly, and use less harmful or non-hazardous alternatives where possible.

## Conclusion

This review details several OCPs, including DDT, HCH, HCB, Cyclodienes, etc. Today, people can be directly or indirectly exposed to OCP through a variety of routes including skin, eyes, nose, mouth, and more. Among them, diet is the most important influencing factor. The negative impact of OCPs toxicity on maternal and infant health is a serious issue that must be addressed. First, OCPs is a type of EED with weak estrogenic activity, and mothers exposed to OCPs before and after pregnancy will cause their accumulation in the body and affect lactation. In addition, OCPs can affect fetal development through the placenta. OCPs can also accumulate in the fat-rich mammary gland, and breast milk is the main source of nutrition for newborns. Transfection of OCPs to infants through breastfeeding brings hidden dangers to the health of infants, including: effects on their height, weight, Effects on the development of the nervous system, effects on the composition of the intestinal flora, effects on the development of the reproductive system, etc. As the most important developmental stage in life, babies should be protected from all toxic and harmful substances. The effects of OCPs on the mother through the mammary gland and on the infant/fetus through breast milk/placenta may raise concerns about how to reduce the health damage of OCPs during pregnancy and the postpartum period and encourage early preventive measures. At present, many methods have been studied for the treatment of OCPs, including the treatment of residual OCPs in water, soil and other environments through various physical, chemical and biological methods. It is also possible to relieve residual OCPs in the body by eating some beneficial foods. Food is the main way that mothers/infants are exposed to OCPs, and heat treatment of them can help reduce the harm of OCPs. However, these methods have their own advantages and disadvantages, and more perfect methods need to be explored to solve this problem. Also, I think public awareness should be raised about the harmful effects of OCPs and how to properly handle them, and where possible use less harmful or harmless alternatives, especially in some developing countries. Further research on OCPs is currently underway, but there are still many unknowns about the mechanism of action of OCPs. We hope this brief review will inform mothers and children health and disease and how to properly manage OCPs.

## Authors Contributions

S-LD conceptualization, S-YQ and X-LX writing original draft preparation, KY and S-LD editing technical review, S-YQ and X-LX visualization, W-ZM and Z-XL supervision. All authors have read and agreed to the published version of the manuscript.

## Funding

This work was supported by National Natural Science Foundation of China (32072722, 32072721, 81860266) and National Transgenic Creature Breeding Grand Project (2016zx08008-003).

## Conflict of Interest

The authors declare that the research was conducted in the absence of any commercial or financial relationships that could be construed as a potential conflict of interest.

## Publisher’s Note

All claims expressed in this article are solely those of the authors and do not necessarily represent those of their affiliated organizations, or those of the publisher, the editors and the reviewers. Any product that may be evaluated in this article, or claim that may be made by its manufacturer, is not guaranteed or endorsed by the publisher.
